# Hypothenar Hammer Syndrome in an Elderly Caucasian Female: A Case Report

**DOI:** 10.7759/cureus.6636

**Published:** 2020-01-12

**Authors:** Jordan T Carter, Michael Polmear, Fernando Herrera, Gilberto Gonzalez

**Affiliations:** 1 Orthopaedics, Texas Tech University Health Sciences Center, El Paso, USA; 2 Plastic Surgery, Medical University of South Carolina, Charleston, USA

**Keywords:** case report, hypothenar hammer syndrome, pseudoaneurysm, surgery, mass

## Abstract

Hypothenar hammer syndrome (HHS) is a vascular disorder characterized by ulnar artery thrombosis or aneurysmal formation. It is most commonly reported in middle-aged males who suffer from repetitive trauma to the palm secondary to occupational or recreational activities. Most cases of HHS can be managed conservatively; however, surgical management is typically indicated for persistent symptoms following conservative measures, imminent vascular compromise, those who fail conservative treatment, or those in imminent danger of rupture. Here we present the case of a right-handed 74-year-old female with HHS who presented with a three-week history of a mass in the hypothenar eminence of the left hand. Reportedly, the mass had appeared slowly and had no associated pain, ischemia of the distal hand, numbness, paresthesia, or changes in the overlying skin. She denied any history of a blunt or penetrating hand injury. The patient was treated surgically by removing a 2.0-cm thrombosed pseudoaneurysm of a collateral branch of the ulnar artery within the left hypothenar eminence. On follow-ups at 1, 2, and 12 weeks postoperatively, the patient's pain was found to be well-controlled. Her normal range of motion was restored, and her digits remained neurovascularly intact. This was an atypical presentation of HHS, and our review of the disorder emphasizes the importance of diagnostic reasoning in rare conditions with unusual presentations of HHS.

## Introduction

Hypothenar hammer syndrome (HHS) is a vascular disorder that predominantly affects the dominant hand of middle-aged men who utilize their hand as a “hammer”, arising from repetitive blunt trauma to the hypothenar eminence or, rarely, from a single traumatic blunt injury to the hypothenar eminence [1-5]. This syndrome is caused by the compression and subsequent thrombosis of the ulnar artery against the hamate bone as it passes through Guyon’s canal [1-3]. Patients typically present with complaints of pain, pallor, cyanosis, cold sensitivity, claudication, and color changes localized to the ulnar digits of the affected hand [1-4]. Angiography is usually used to identify the occlusion and define the anatomy, especially if surgical intervention is required. Due to the rarity of this syndrome, no prospective studies have been published so far to identify the most appropriate treatment strategy [1]. Management includes smoking cessation, activity modification, pharmacological therapy, direct thrombin injection, transarterial coiling, and operative intervention for failed conservative management, impending aneurysmal rupture, or ischemia [4-8]. Here we review the literature on HHS and discuss an atypical presentation encountered at our institution.

## Case presentation

We present the case of a right-handed, 74-year-old female who presented with a three-week history of a mass in the hypothenar eminence of the left hand. According to the patient, the mass had appeared slowly and had no associated pain, ischemia of the distal hand, numbness, paresthesia, or changes in the overlying skin. She denied any history of a blunt or penetrating hand injury. The patient was a lifelong nonsmoker and had a past medical history significant for right breast cancer (status: post lumpectomy and radiation), with postoperative right upper extremity lymphedema.

**Figure 1:**
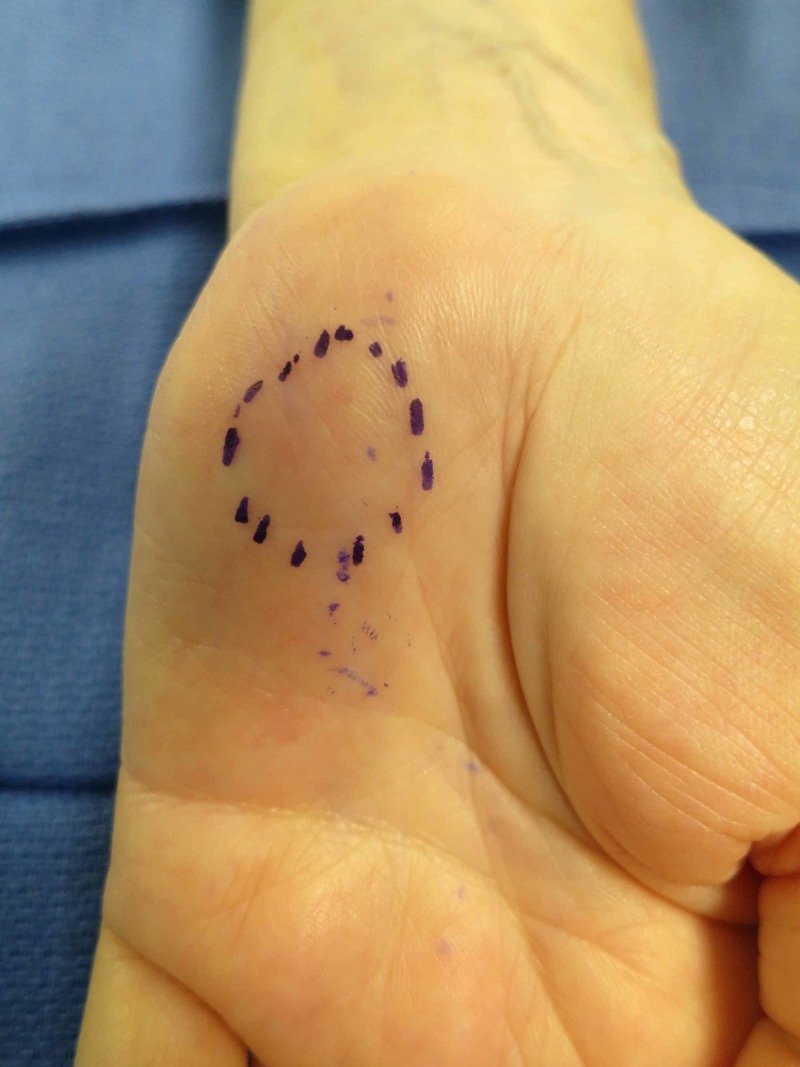
Enlarging mass seen on the patient’s hypothenar eminence

A physical exam of the left upper extremity showed a non-tender, non-pulsatile, firm 1.5 x 1.5-cm subcutaneous mass within the hypothenar eminence at the hook of the hamate (Figure 1). The patient exhibited no neurovascular changes in the hand and demonstrated a normal Allen’s test. Radiographic evaluation showed osteoarthritis of the distal and proximal interphalangeal joints and no acute osseous abnormalities, dislocations, heterotopic ossification, or bone coalitions. An MRI showed a 1.2 x 1.0 x 1.7-cm thrombosed pseudoaneurysm of the distal ulnar artery at the level of Guyon’s canal. Subsequent arterial duplex sonography revealed patent left upper extremity arteries with standard anatomy, including a patent palmar arch. 

**Figure 2:**
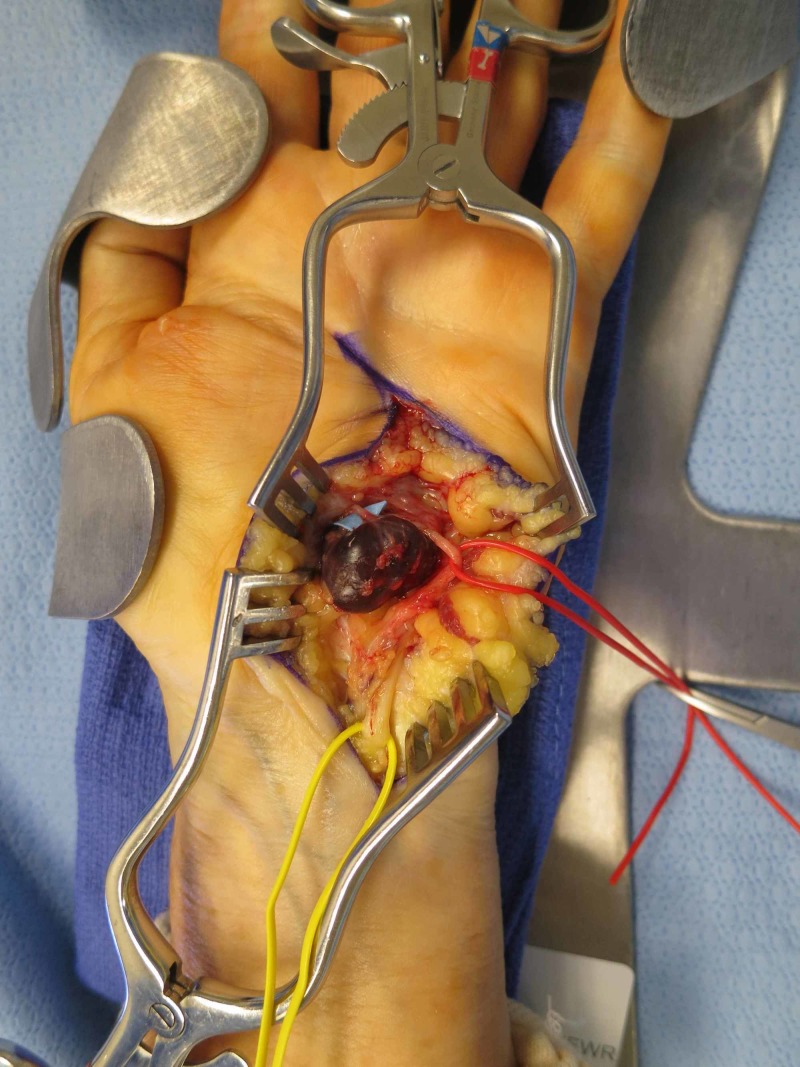
Intra-operative view

**Figure 3:**
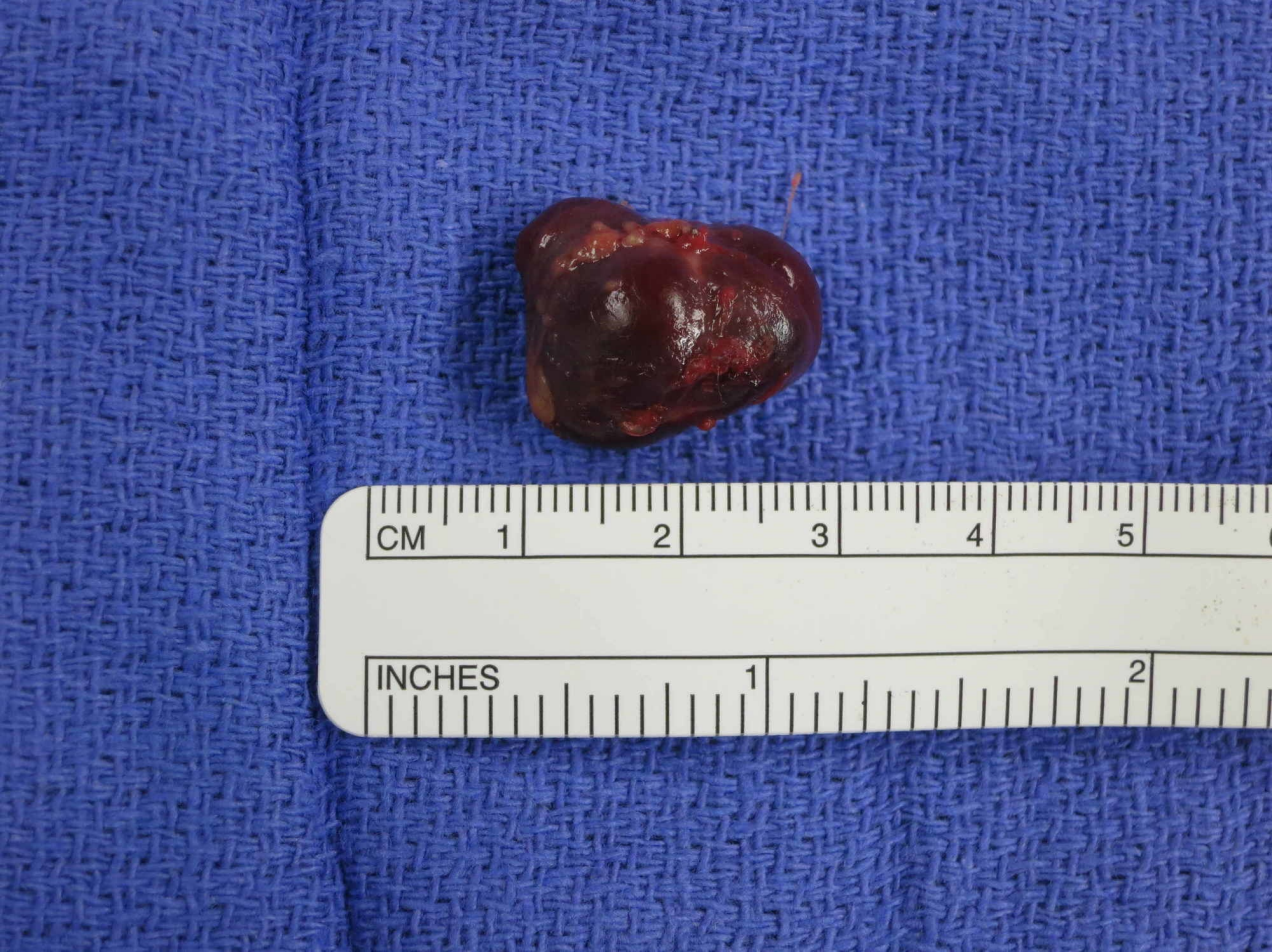
Resected pseudoaneurysm

The patient was offered pseudoaneurysm resection to prevent possible rupture and ischemia, and she chose to proceed with operative management. During the procedure, the patient was prepped and draped; a tourniquet was inflated to 250 mmHg; an incision was made at Guyon’s canal. A 2.0-cm mass was located radial to the neurovascular bundle, extending from a collateral branch of the ulnar artery after the bifurcation into the deep and superficial branches (Figure 2). The mass was dissected and clipped at its origin, after which the tourniquet was released to confirm that flow through the ulnar artery was intact. The mass was then excised, and the subsequent pathologic examination was consistent with a thrombosed pseudoaneurysm (Figure 3). 

**Figure 4:**
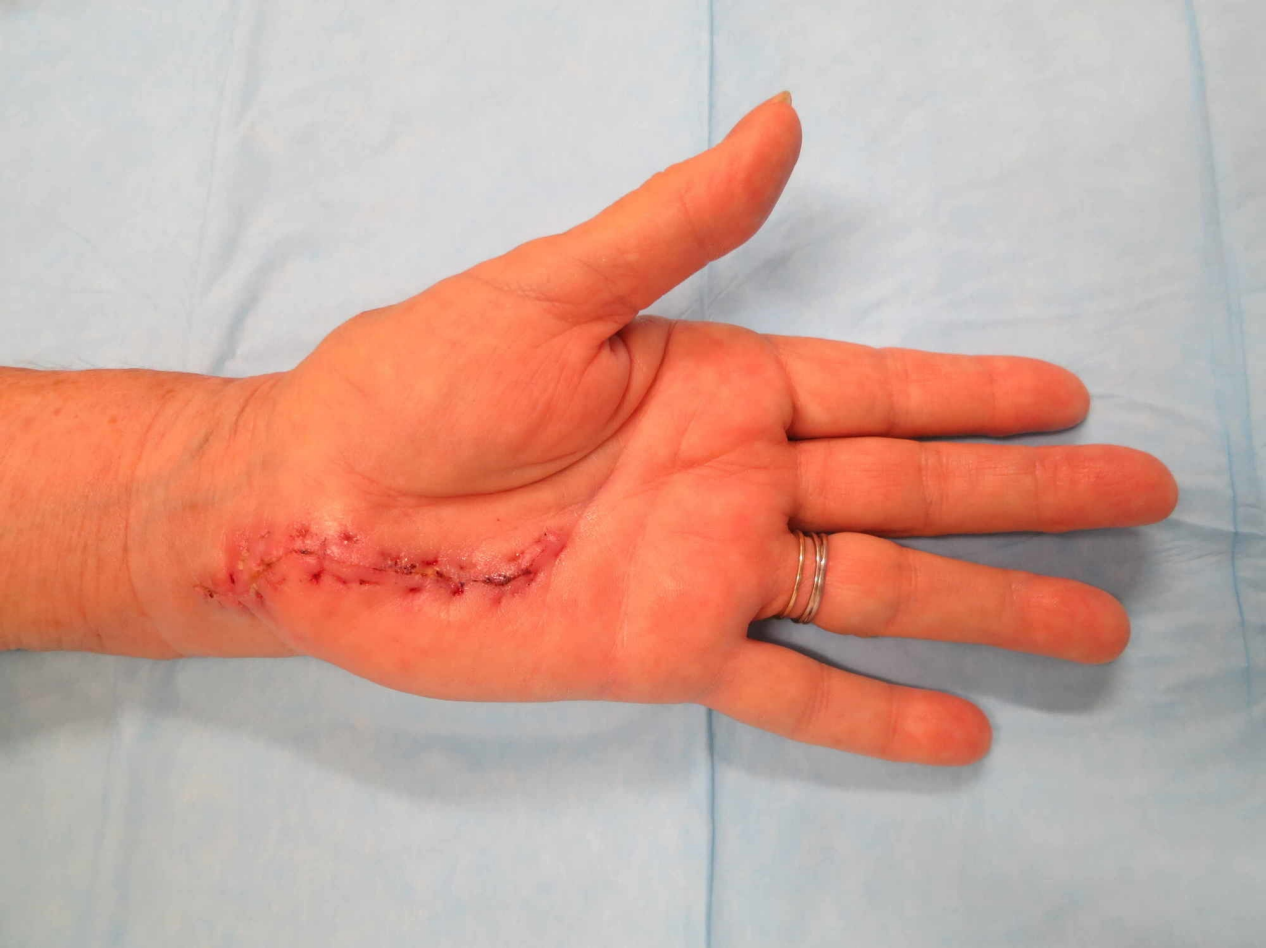
Post-surgical incision

On follow-ups at 1, 2, and 12 weeks postoperatively, the patient presented with the scar seen in Figure 4. Her pain was found to be well-controlled. Her normal range of motion was restored, and her digits remained neurovascularly intact. 

## Discussion

Causes

HHS usually results from compression of the ulnar artery just distal to Guyon’s canal [1,3,9]. This course of the ulnar artery narrows at the proximal aspect of the hypothenar eminence where it is surrounded by the pisiform, hamate, and pisohamate ligament [5]. As the ulnar artery emerges, it branches into the superficial and deep palmar arches [1]. The superficial branch traverses the hypothenar muscles and then penetrates the palmar aponeurosis [1]. Prior to penetration, this segment can be compressed against the adjacent hook of hamate as a consequence of repetitive trauma or a single injury [1,2,3,5,10]. This syndrome was first recognized by Conn et al. who coined the term “hypothenar hammer syndrome” [11].

Most cases of HHS are caused by repetitive microtraumas that are thought to stimulate vasospasms causing intimal hyperplasia [12]. This can lead to platelet aggregation, thrombus formation, and occasionally, aneurysmal formation [12,13]. This cycle is propagated as thrombus formation, causing vasospasm of nearby vasculature, which further decreases perfusion [9,12]. This repetitive trauma and insult cause thickening, fibrosis, and scarring [9]. If an aneurysm forms, the nearby ulnar nerve can be compressed, resulting in pain and paraesthesia [1,3,5]. It has been theorized that the limited incidence of HHS, despite a large number of individuals who repetitively use their hand with blunt microtrauma, could be secondary to anatomical variability, subclinical disease, and underlying abnormal arterial histology [1-3,14].

Presentation of hypothenar hammer syndrome

This syndrome is typically seen in laborers, such as carpenters, butchers, and mechanics who use their hand as a “hammer” as part of their profession. HHS has also been associated with sports and hobbies requiring repetitive compression or blunt trauma over the hypothenar eminence. Although rare, HHS has also been documented as resulting from a singular blunt or penetrating trauma to the hypothenar eminence [2,4-7,9,14]. HHS typically presents as pain, pallor, cyanosis, coolness, numbness, and color changes in the ulnar digits secondary to arterial insufficiency [1-5]. Ulceration and gangrene can be present in extreme cases [2]. Occasionally, an associated aneurysm can present as a palpable, pulsatile mass [1,3,4].

HHS is typically observed in the ulnar digits of the dominant hand in males [1,3-7,14]. The thumb is typically spared, allowing for differentiation from Raynaud’s phenomenon [1,4]. The average age of onset is in the fifth decade of life [1,4,7]; however, cases have been reported in older individuals [15]. Most patients cite an average duration of symptoms of two-and-a-half months, highlighting the typical subacute development of HHS [7].

As HHS is likely under-reported, its incidence is hard to determine. One study found 22 cases (1.7%) in 1,300 patients referred to their center for hand ischemia [14]. A larger study found that of the 4,148 patients referred for Raynaud’s phenomenon, 47 (1.1%) cases were actually HHS [1]. 

Diagnosis

Diagnosis of HHS requires clinical suspicion and can be confirmed via different imaging modalities, such as the characteristic “ying-yang sign” on ultrasound or CT angiography [5,6]. On physical exam, an abnormal Allen’s test [2], suggesting a degree of occlusion in the ulnar artery, and the unilateral nature of HHS helps differentiate it from similarly presenting pathologies, such as Raynaud’s phenomenon [9]. Although HHS can result in aneurysmal formation, the presence of an aneurysm should also prompt consideration of congenital, mycotic, and penetrating trauma etiologies [16].

Catheter-based angiography remains the gold standard to identify the lesion and nearby anatomy, especially if surgical intervention is being considered [1]. CT angiography has high sensitivity (90-95%) and specificity (98-100%) for HHS [1]. Advanced imaging can help to elucidate nearby structures and underlying bony or muscular abnormalities [1,4,7,9,12,17,18]. Regardless of imaging choice, the proximal segments of the nerve must also be examined to ensure that a proximal obstruction is not present [17].

Treatment

Due to the relatively rare nature of HHS, there is no consensus on the best management of the condition [1,3,7]. Conservative means consist of patient education on hand protection, avoiding cold and exacerbating activities and, perhaps most importantly, counseling on smoking cessation. Patients who continue to smoke typically fail both conservative and surgical interventions [3,7]. Pharmacological options include calcium channel blockers, antiplatelet or anticoagulant agents, and pentoxifylline [1,8,10]. Intra-arterial thrombolytics can be offered on the premise of reducing distal embolic events [19]. In one study of four patients with HHS, three experienced improvements after intra-arterial thrombolysis and there were no adverse bleeding events [20]. Conversely, the use of ultrasound-guided thrombin injection has been used with mixed results in the treatment of small vessel pseudoaneurysms including HHS [5,6]. Conservative treatment is usually effective, and a small case series have demonstrated improvement in as many as 83% of patients with conservative treatment [7,8]. However, recurrence is a concern and has been demonstrated in up to 27.7% of conservatively managed cases, namely in patients who continued smoking throughout treatment [6,7]. Surgery is typically indicated for ischemia and vascular damage without adequate collateral circulation [4,6,8]; and options include arterial ligation, resection of the thrombosed or aneurysmal segment with end-to-end anastomosis, or arterial resection with graft reconstruction [1,6-8,10].

## Conclusions

HHS is a relatively rare syndrome, typically presenting in middle-aged males who undergo repetitive trauma to the ulnar side of the palm. This case represented a unique presentation of HHS in a 74-year-old female with no history of trauma or repetitive activity to the hypothenar eminence. Also novel to this presentation was the occurrence of a thrombosed pseudoaneurysm in the non-dominant hand and the lack of associated symptoms, leading us to ponder on the etiology of this patient’s condition given the lack of any typical causative factors. This case, in particular, emphasizes the importance of diagnostic reasoning in rare presentations of HSS. Due to the unique presentation seen in the case above, the diagnosis could have been missed at the expense of morbidity to the patient. 
